# Autoimmunity and Cytokine Imbalance in Inherited Epidermolysis Bullosa

**DOI:** 10.3390/ijms17101625

**Published:** 2016-09-24

**Authors:** Susanna Esposito, Sophie Guez, Annalisa Orenti, Gianluca Tadini, Giulietta Scuvera, Laura Corti, Alessia Scala, Elia Biganzoli, Emilio Berti, Nicola Principi

**Affiliations:** 1 Pediatric Highly Intensive Care Unit, Department of Pathophysiology and Transplantation, Università degli Studi di Milano, Fondazione IRCCS Ca’ Granda Ospedale Maggiore Policlinico, Milan 20122, Italy; sguez_2000@yahoo.com (S.G.); annalisa.orenti@unimi.it (A.O.); gtadinicmce@unimi.it (G.T.); giulietta.scuvera@hotmail.it (G.S.); a.scala19@gmail.com (A.S.); nicola.principi@unimi.it (N.P.); 2 Dermatology Unit, Department of Pathophysiology and Transplantation, Università degli Studi di Milano, Fondazione IRCCS Ca’ Granda Ospedale Maggiore Policlinico, Milan 20122, Italy; cortilaura@hotmail.com (L.C.); emilio.berti@unimib.it (E.B.); 3 Unit of Medical Statistics, Biometry and Bioinformatics “G.A. Maccacaro”, Department of Clinical Sciences and Community Health, Università degli Studi di Milano, Milan 20122, Italy; elia.biganzoli@unimi.it

**Keywords:** autoimmunity, Birmingham Epidermolysis Bullosa Severity (BEBS) score, epidermolysis bullosa, inflammatory response, inherited epidermolysis bullosa

## Abstract

In order to evaluate the serum anti-skin autoantibodies and cytokine concentrations in patients with different epidermolysis bullosa (EB) types and severity, 42 EB patients and 38 controls were enrolled. Serum anti-skin antibodies were significantly higher in the patients than in the controls (*p* = 0.008, *p* < 0.001, *p* < 0.001, *p* < 0.001 and *p* < 0.001 for desmoglein 1 (DSG1) desmoglein 3 (DSG3), bullous pemphigoid 180 (BP180), BP230 and type VII collagen (COL7), respectively). The same trend was observed for interleukin (IL)-1β, IL-2, IL-6, IL-10, tumor necrosis factor-β, and interferon-γ (*p* < 0.001, *p* < 0.001, *p* < 0.001, *p* = 0.008, *p* < 0.001 and *p* = 0.002, respectively). Increases in anti-skin antibodies and cytokine concentrations were higher in patients with recessive dystrophic EB than in those with different types of EB, in generalized cases than in localized ones, and in patients with higher Birmingham Epidermolysis Bullosa Severity (BEBS) scores than in those with a lower score. The BEBS score was directly correlated with BP180, BP230, COL7 (*p* = 0.015, *p* = 0.008 and *p* < 0.001, respectively) and IL-6 (*p* = 0.03), whereas IL-6 appeared significantly associated with DSG1, DSG3, BP180, BP230 and COL7 (*p* = 0.015, *p* = 0.023, *p* = 0.023, *p* = 0.015 and *p* = 0.005, respectively). This study showed that autoimmunity and inflammatory responses are frequently activated in EB, mainly in severe forms, suggesting the use of immunosuppressive drugs or biologicals that are active against pro-inflammatory cytokines to reduce clinical signs and symptoms of disease.

## 1. Introduction

Inherited epidermolysis bullosa (EB) encompasses a heterogeneous cluster of rare, genetically transmitted disorders comprising several blistering skin diseases with a monogenic basis and either autosomal dominant or recessive inheritance [1]. Initially, the EB types were classified according to the level in which blisters developed and were differentiated into EB simplex (EBS; fragility and blistering confined to the epidermis), junctional EB (JEB; blisters developed within the lamina of the skin basement membrane zone) and dystrophic EB (DEB; blister occurring within the uppermost dermis). A fourth type, Kindler syndrome, comprised all cases that were characterized, together with specific clinical characteristics (mainly photosensitivity), by blisters developing in multiple levels in the skin basement membrane area [1]. However, several clinical subtypes have been identified and defined in each group according to phenotypic and genotypic characteristics. For this reason, in recent years, a new approach to classification—“onion skinning”—has been introduced [2]. This approach takes into account sequentially the major EB type accordingly with the old classification, phenotypic characteristics, the mode of inheritance, the targeted protein and its relative expression in skin, the gene involved, and the type(s) of mutation present and, when possible, specific mutation(s) and their locations(s). The Birmingham Epidermolysis Bullosa Severity (BEBS) scale has been validated for the classification of EB severity. It assesses the area of damaged skin, the involvement of nails, mouth, eyes, larynx and esophagus, the scarring of hands, skin cancer, chronic wounds present for at least six months, alopecia and nutritional compromise. Area is allocated 50 points and the 10 other items 5 points each, providing a maximum score of 100 [3].

EB is caused by mutations of genes encoding for specific structural skin proteins. For example, mutations of the *KRT5* and *KRT14* genes encoding for type I and type II keratin intermediate filaments are responsible for EBS [4], whereas mutations in the *COLT7A1* gene encoding for type VII collagen (COL7) are the cause of DEB [5]. However, genotype–phenotype correlations were not always shown, and subjects with the same genetic mutations were frequently found to have very different clinical characteristics. These observations led us to consider the possibility that in the genesis of EB over the alterations caused by genetic mutations, other factors could be significant. Several lines of evidence seem to support this hypothesis. First, some cases of EB are complicated by autoimmune extracutaneous diseases [6,7,8,9,10,11]. Moreover, in several patients with EB, high levels of anti-skin antibodies, proportional to the severity of the disease, could be found [12,13]. Finally, a significant cytokine imbalance was demonstrated in EB, suggesting the presence of a systemic inflammatory disorder [13]. All of these findings seem to indicate that EB is a systemic disorder in which several factors play a role in determining the development and severity of the different EB types. Confirmation of this hypothesis might have a relevant impact on the diagnosis and treatment of EB. The concentration of anti-skin antibodies and pro-inflammatory cytokines may have a role in disease severity and could represent a useful tool for establishing EB prognosis. Moreover, drugs useful for controlling autoimmunity and inflammatory disorders might be treatment options for a complex, sometimes very severe, disease for which no satisfactory therapy is presently available. This study was planned to determine serum anti-skin autoantibodies and cytokine concentrations in a group of subjects with different EB types in order to study the correlations with EB phenotype and disease severity.

## 2. Results

A total of 42 EB patients (24 males, 57.1%; mean age ± standard deviation (SD), 15.4 ± 14.3 years) and 38 controls (20 males, 52.6%; mean age ± SD, 15.5 ± 14.0) were enrolled. Of the patients, 13 (30.9%) were classified as EBS, 22 (52.4%) as DEB (19 RDEB and 3 as dominant DEB (DDEB)), 5 (11.9%) as JEB, and 2 (4.8%) as Kindler syndrome.

Table 1 shows the serum levels of anti-skin antibodies and cytokines in all of the EB patients and controls. All anti-skin antibodies were significantly higher in the EB patients than in the controls (*p* = 0.008, *p* < 0.001, *p* < 0.001, *p* < 0.001 and *p* < 0.001 for desmoglein 1 (DSG1), desmoglein 3 (DSG3), bullous pemphigoid 180 (BP180), BP230 and type VII collagen (COL7), respectively). The same trend was evidenced for many cytokines, in particular interleukin (IL)-1β, IL-2, IL-6, IL-10, TNF-β, and IFN-γ (*p* < 0.001, *p* < 0.001, *p* < 0.001, *p* = 0.008, *p* < 0.001 and *p* = 0.002, respectively). The serum levels of IL-8 and IL-12 were also higher in the EB patients than in the controls, although the difference did not reach statistical significance (*p* = 0.197 and *p* = 0.056, respectively). Only the IL-4 and TNF-α serum levels did not differ between the groups.

Table 2 shows the comparisons of the cytokine and antibody serum concentrations between RDEB and all other types of EB cases as well as between all other types of EB and the controls. The EB cases not classified as RDEB were considered together because a preliminary evaluation did not show any significant difference between the various types. The data indicated that the increase in the studied anti-skin antibody concentrations was higher in patients with RDEB than in those with other types of EB, although in the other EB patients the concentrations of the studied variables remained higher than in the healthy controls. The differences between the RDEB and other EB patients were statistically significant for all studied anti-skin antibodies (*p* < 0.001, *p* = 0.019, *p* = 0.015 and *p* < 0.001 for DSG3, BP180, BP230 and COL7, respectively), with the exception of anti-DSG1 (*p* = 0.066) after correction with the Benjamini-Hochberg procedure. However, for anti-DSG1, the mean serum concentrations in patients with RDEB were approximately double those in patients with other EB types. The median serum concentrations of the studied cytokines did not significantly vary between RDEB and other EB patients, although they were higher in RDEB than in other EB cases. However, serum IL-1β, IL-2, IL-6, and TNF-β concentrations were significantly higher in the EB patients with other EB types than in the controls (*p* = 0.027, *p* = 0.008, *p* = 0.008 and *p* < 0.001, respectively).

Table 3 shows the results of the studied variables for patients with localized or generalized EB and in the controls. Anti-skin antibodies were significantly higher in generalized cases than in localized EB cases (*p* = 0.048, *p* = 0.026, *p* = 0.005, *p* < 0.001 and *p* = 0.001 for DSG1, DSG3, BP180, BP230 and COL7, respectively). However, the patients with localized lesions had higher anti-skin antibody levels than the controls, although significant differences were observed only in COL7 (*p* = 0.036). Regarding serum cytokine concentrations, although IL-1β, IL-2, IL-6, IL-8 and TNF-α were higher in the patients with generalized EB than in those with localized forms, no significant difference was observed between the groups. However, the controls showed significantly lower values of IL-1β, IL-2, IL-6, TNF-β and IFN-γ than did the patients with localized EB (*p* = 0.048, *p* = 0.03, *p* = 0.036, *p* < 0.0001 and *p* = 0.036, respectively).

Table 4 shows the comparison of the studied variables in EB patients with high and low BEBS scores. The results indicated that the anti-skin antibodies were significantly higher in patients with higher BEBS scores than in those with lower values (*p* = 0.008, *p* = 0.0012, *p* = 0.005, *p* < 0.001 and *p* < 0.001 for DSG1, DSG3, BP180, BP230 and COL7, respectively). However, the EB patients with lower BEBS scores had higher anti-skin antibody levels than did the healthy controls, with significant differences for DSG3, BP180, BP230 and COL7 (*p* = 0.033, *p* = 0.016, *p* = 0.022 and *p* < 0.001, respectively). Moreover, although IL-1β, IL-2, IL-6, TNF-α, TNF-β and IFN-γ were higher in the EB patients with higher BEBS scores than in those with lower values, only differences in IL-6 resulted statistically significant (*p* = 0.022) after correction with the Benjamini-Hochberg procedure. However, the EB patients with a lower BEBS score showed significantly higher IL-1β, IL-2, IL-6, IL-10, IL-12, TNF-β and IFN-γ than did the controls (*p* = 0.015, *p* = 0.004, *p* = 0.004, *p* = 0.038, *p* = 0.038, *p* < 0.001 and *p* = 0.021, respectively).

Table 5 summarizes the correlation between anti-skin antibodies, serum cytokine levels, and the BEBS scores in EB patients. The BEBS score was directly correlated with BP180, BP230, COL7 (*p* = 0.015, *p* = 0.008 and *p* < 0.001, respectively), and IL-6 (*p* = 0.03). In addition, IL-6 appeared to be significantly associated with DSG1, DSG3, BP180, BP230, and COL7 (*p* = 0.015, *p* = 0.023, *p* = 0.023, *p* = 0.015 and *p* = 0.005, respectively) (Table 6).

## 3. Discussion

This study demonstrates that autoimmunity and inflammatory responses are frequently activated in EB with a possible role in conditioning clinical manifestations of the disease. Several serum anti-skin antibodies were significantly higher in patients than in healthy controls, and the increases were strictly related to the severity of the disease and to the inflammatory response (mainly evidenced by the IL-6 increase). Patients with RDEB (i.e., the EB type with the most severe clinical manifestations), those with generalized EB and those with a higher BEBS score were the patients who showed the highest increase in serum anti-skin antibodies and cytokine concentrations.

Our findings extend what was reported by Tampoia et al. who, studying serum concentrations of BP180, BP230 and COL7 antibodies, found that the levels were significantly higher in patients with RDEB than in EBS patients, and the values were correlated with EB severity [12]. In our study population, in addition to antibodies against BP180, BP230 and COL7, antibodies against DSG1 and DSG3 were evaluated. These proteins are members of the cadherin cell adhesion molecule superfamily and are major components of the cell–cell junctions that help resist shearing forces and are found in high concentrations in cells subject to mechanical stress [14]. The pathogenic role of these antibodies has previously been shown in experimental animals and in humans. Anti-COL7 antibodies injected into mice produce a blistering disease [15]. Circulating autoantibodies against the dermoepidermal junction are crucial to the pathogenesis of acquired epidermolysis bullosa (EBA), an autoimmune disease clinically resembling EB [16]. Increases in serum concentrations of BP180 and BP130 are characteristically present in patients with bullous pemphigoid [17]. Finally, antibodies against DSG1 and DSG3 have been identified in pemphigus vulgaris or foliaceus [18]. Even though it cannot be excluded that the higher levels of anti-skin antibodies are the consequence of the deterioration of the skin condition, the similarities with what has been demonstrated in several acquired autoimmune skin diseases seems to indicate that, in EB, activation of autoimmunity is a possible cause of further skin damage. It could be supposed that, in EB, the skin and mucosal damage depends on genetic factors but is aggravated by the activation of an immune process that could take, in certain cases, a major role in defining the severity of the clinical manifestations. In animal models, it has been shown that COL7 is present in gastrointestinal tissue, which can be damaged by anti-COL7 antibodies [19]. Moreover, in humans with EB, several autoimmune diseases can develop. Gastrointestinal and renal immune-mediated complications have been widely described, including celiac disease, amyloidosis, post-infectious glomerulonephritis and IgA nephropathy [6,7,8,9,10]. Finally, in acquired EB, inflammatory bowel diseases have been described in approximately 30% of cases [20].

Several hypotheses have been proposed to explain why, in inherited EB, an autoimmune mechanism aggravates the basal disease [12], but the precise triggers for the breakdown of self-tolerance and the subsequent events leading to the induction of pathogenic autoimmune responses remain undefined. Considering that children with the more severe EB phenotype usually have a greater number of skin infections, it could be supposed that an altered microbial diversity may cause a worsening of skin inflammation and autoimmunity. Some data collected in this study and other studies regarding cytokine imbalance in patients with EB suggest that the induction of a chronic inflammatory response could explain, at least in part, the activation of autoimmunity and the deterioration and extensions of the basal EB lesions. Cytokines play a pivotal role in the pathogenesis of autoimmune diseases, and increased serum levels of a number of cytokines are associated with the development and progression of systemic autoimmune diseases [21,22]. In particular, pro-inflammatory cytokines contribute to the initiation and propagation of autoimmune inflammation, although the role of each cytokine is significantly influenced by the concentration, the stage of disease during which the cytokine is secreted and its combination with other cytokines, particularly those with an anti-inflammatory effect [22]. IFN-γ is considered a prototypic proinflammatory biomarker of autoimmune inflammation, and its administration to mice results in accelerated autoimmune diseases [23]. In experimental animals, persistent release of IL-1 family members has been associated with the occurrence of severe systemic cardiovascular diseases and metabolic abnormalities, including aberrant vascular wall remodeling with aortic stenosis, cardiomegaly, impaired limb, tail circulation, fatty tissue loss and systemic amyloid deposition in multiple organs with liver and kidney dysfunction [24]. Increases in IL-6 could have a relevant effect because this cytokine promotes Th17 differentiation. Early studies in animal models and in patients with multiple sclerosis and rheumatoid arthritis have shown that IL-17 produced by Th17 cells plays a role in inducing autoimmune diseases, mainly by activating the innate immune response [25,26]. Moreover, Lei et al. reported that Th17 cells participate in the pathogenesis of skin and lung fibrosis by enhancing fibroblast proliferation and cytokine production in a bleomycin-induced murine model of systemic sclerosis [27].

In this study, most of the cytokines with pro-inflammatory activity (i.e., IL-1β, IL-2 IL-6, TNF-β and INF-γ) were significantly higher in patients with EB than in healthy controls, and the levels were higher in RDEB patients than in other EB patients. Moreover, IL-6 serum levels were significantly correlated with EB extension and severity and with anti-skin antibody concentrations. The increase in pro-inflammatory cytokines seems to confirm that EB is a systemic disease, explaining the extracutaneous involvement frequently observed in EB. The amount of the inflammatory response seems to be critical in conditioning autoimmune activity and consequently, for the severity of the disease. On the other hand, increased level of several cytokines, including IL-1 and IL-6 not substantially different from those found in this study, have been found in both sera and blister fluid of patients and experimental animals with acquired EB. Moreover, in most cases, disease activity correlates with cytokine levels, confirming the pathogenic role of these proteins in the determination of skin lesions [28].

In agreement with the results of the study by Annichiarico et al. [13] but in contrast with the data published several years ago by Chopra et al. [29,30], an increase in the IL-2 serum concentration in EB cases regardless of disease severity was shown in this study. IL-2 signaling is essential for the development, function and homeostasis of regulatory T cells, and increased levels of this cytokine have been shown in skin diseases, such as psoriasis [31]. Moreover, the reduction of IL-2 together with the reduction of the levels of other pro-inflammatory cytokines has been found to be associated with a significant improvement of skin diseases [31]. Although further data regarding the actual role of IL-2 in EB are needed to explain the discordant results, the increase in IL-2 could represent a further indication of the cytokine imbalance present in EB cases that can lead to autoimmunity and systemic body involvement.

## 4. Materials and Methods

### 4.1. Study Population and Recruitment

The study was performed between 1 April 2015 and 30 September 2015, and involved all of the patients with EB regularly followed by the Centre for Epidermolysis Bullosa at Fondazione IRCCS Ca′ Granda Ospedale Maggiore Policlinico, Università degli Studi di Milano, Milan, Italy. Clinical phenotype, electronic microscopy on samples obtained using skin biopsy and genetic mutation analysis supported the diagnosis in accordance with the Third International Consensus Meeting on Diagnosis and Classification of EB [32].

Upon enrollment, the demographic characteristics and medical history of the patients were systematically recorded using standardized written questionnaires. EB severity was assessed using the BEBS score [3]. As a control group, otherwise healthy individuals with a similar age and gender distribution without EB, without chronic underlying diseases and without a history of skin disorders were enrolled.

A 5-mL whole-blood sample was taken from all enrolled patients and controls, for serum cytokine and anti-skin antibody measurements.

The protocol was approved by the Ethics Committee of the Fondazione IRCCS Ca′ Granda Ospedale Maggiore Policlinico, Milan, Italy (number 359, determination 1409-2015) and written informed consent was obtained from the enrolled subjects. For individuals younger than 18 years of age, informed consent was signed by both parents or a legal guardian. Children ≥8 years were required to give their written assent before entering the study.

### 4.2. Cytokine Measurement

Serum concentrations of IL-1β, IL-2, IL-4, IL-6, IL-8, IL-10, IL-12, tumor necrosis factor (TNF)-α, TNF-β, and interferon (IFN)-γ were measured using a human instant enzyme-linked immunosorbent assay (ELISA) (Ray Biotech, Atlanta, GA, USA), according to the manufacturer′s instructions. Patient and control sera were added to each well of a microwell plate. The sera were incubated for 2.5 h at room temperature. After washing to remove any unbound proteins, a tetramethylbenzidine substrate was added and incubated for 10 min at room temperature. The acid solution was then added to each well to terminate the enzyme reaction and stabilize the color development. The value of each sample was obtained by comparing the optical density of the sample with the optical density of the calibrator and was expressed in pg/mL. The absorbance was measured at 450 nm by an ELlSA reader (Bio-Rad Laboratories, Hercules, CA, USA).

### 4.3. Anti-Skin Autoantibodies Detection

Antibodies against desmoglein 1 (recombinant purified DSG1), desmoglein 3 (recombinant purified DSG3), bullous pemphigoid 180 (recombinant purified BP 180NC16a antigen), BP230 (recombinant purified BP230-N and BP230C antigens) and type VII collagen (COL7; recombinant human NC1 and NC2 proteins) were determined by a commercial ELISA method (MBL International, Nagoya, Japan), according to the manufacturer′s instructions. Briefly, calibrator, patient, and control sera were diluted (1:100), added to each well of a microwell plate coated with purified antigens, and incubated for 1 h at room temperature. After washing with PBS Tween 20, 100 µL of conjugated solution containing horseradish peroxidase-conjugated goat-anti-human IgG antibodies were added to each well and incubated for 1 h at room temperature. After extensive washing, a tetramethylbenzidine substrate was added and incubated for 30 min at room temperature. The acid solution was then added to each well to terminate the enzyme reaction and stabilize the color development. The value of each sample was obtained by comparing the optical density of the sample with the optical density of the calibrator and was expressed in U/mL. The absorbance was measured at 490 nm by an ELlSA reader (Bio-Rad Laboratories, Hercules, CA, USA).

### 4.4. Statistical Analysis

Serum levels of anti-skin antibodies (DSG1, DSG3, BP180, BP230 and COL7) and the studied cytokines (IL-1β, IL-2, IL-4, IL-6, IL-8, IL-10, IL-12, TNF-α, TNF-β, and IFN-γ) were reported for the patients and controls in terms of the median and range because their distributions were asymmetric and outliers were present. To evaluate if the differences observed in the serum levels between the patients and controls were statistically significant, a Wilcoxon rank sum test was performed, because this method enables comparisons between the medians of two distributions. Because of the presence of subjects with the same serum levels (ties), an exact version of the Wilcoxon rank sum test with a correction factor was adopted [33]. Furthermore, *p*-values were adjusted for multiple testing using the false discovery rate method (with the Benjamini-Hochberg procedure).

The same analysis (computing the median and range, performing the exact Wilcoxon rank sum test and controlling for multiple testing) was performed for comparisons between recessive DEB (RDEB; i.e., the most severe EB type) and all other types of EB [2], between localized and generalized EB according to Fine et al. classification [2], and between severe and mild EB according to BEBS score (i.e., patients with a BEBS score over the second tertile, corresponding to 23.5, were considered severe EB) [3].

The relationships between BEBS scores and anti-skin antibodies or cytokines, and the relationships between the IL-6 serum levels and anti-skin antibodies concentrations, were evaluated by means of Spearman′s rank correlation coefficient.

All statistical analyses were performed using R software (Vienna, Austria), version 3.2.2, with the coin package added.

## 5. Conclusions

The data collected in this study demonstrated that autoimmunity and inflammatory responses are frequently activated in EB, adding new information on EB pathogenesis. Moreover, this study seems to indicate new therapeutic approaches for a disease for which there are no effective therapies. The use of immunosuppressive drugs or biologicals that are active against pro-inflammatory cytokines, which are the most important cause of inflammation and autoimmunity, might reduce clinical signs and symptoms of disease and significantly improve the quality of life of EB patients. Ad hoc studies, including a detailed analysis of the potential adverse events (including development of infections) strictly related to the use of these drugs, are urgently needed as an attempt to face a very difficult to treat disease such as EB.

## Figures and Tables

**Table 1:** Serum anti-skin antibodies and cytokine concentrations in patients with inherited epidermolysis and healthy controls.

Parameter	Total *n* = 80	Controls *n* = 38	EB Cases *n* = 42	*p*-Value	Corrected *p*-Value *
Anti-skin antibodies, U/mL					
DSG1	2.72 (0–38.83)	2.12 (0–15.09)	3.83 (0.18–38.83)	0.005	0.008
DSG3	2.8 (0–40.4)	1.58 (0.08–8.38)	3.72 (0–40.4)	<0.001	<0.001
BP180	3.9 (0–118.8)	1.82 (0–34.41)	7.1 (0.5–118.8)	<0.001	<0.001
BP230	3.55 (0.22–66)	1.68 (0.22–26.74)	4.75 (0.5–66)	<0.001	<0.001
COL7	0.8 (0–41.05)	0.26 (0–3.99)	1.92 (0–41.05)	<0.001	<0.001
Cytokines, pg/mL					
IL-1β	5.79 (0.88–192.67)	2.75 (0.88–189.66)	16.62 (1.16–192.67)	<0.001	<0.001
IL-2	61.55 (8.01–3992.66)	31.61 (8.01–2400)	111.23 (18.76–3992.66)	<0.001	<0.001
IL-4	0.96 (0.79–4.5)	0.95 (0.8–2.07)	0.96 (0.79–4.5)	0.319	0.342
IL-6	15.12 (4.46–1000)	8.54 (4.46–1000)	33.44 (5.32–1000)	<0.001	<0.001
IL-8	39.92 (12.96–939.06)	27.96 (12.96–939.06)	45.09 (13.14–449)	0.171	0.197
IL-10	28.88 (5.86–250)	21.12 (5.86–250)	32.98 (8–250)	0.006	0.008
IL-12	1.46 (1–9.21)	1.36 (1–7.29)	1.52 (1.14–9.21)	0.045	0.056
TNF-α	176.1 (128.17–4800)	176.1 (128.17–1351.09)	175.12 (137.66–4800)	0.385	0.385
TNF-β	1538.24 (802.84–36,000)	1267.11 (802.84–3764.79)	1737.07 (1101.8–36,000)	<0.001	<0.001
IFN-γ	220.68 (73.56–5000)	176.34 (73.56–910)	330.16 (79.18–5000)	0.001	0.002

BP180: antibodies against bullous pemphigoid 180; BP230: antibodies against bullous pemphigoid 230; COL7: type VII collagen; DSG1: antibodies against desmoglein 1; DSG3: antibodies against desmoglein 3; EB: inherited epidermolysis bullosa; IFN: interferon; IL: interleukin; TNF: tumor necrosis factor. Median values (range in parentheses). * *p*-Values were adjusted for multiple testing using the false discovery rate method (with the Benjamini-Hochberg procedure).

**Table 2:** Comparison between different inherited epidermolysis bullosa types and healthy controls.

Parameter	Total EB Cases *n* = 42	Other EB Types *n* = 23	RDEB *n* = 19	*p*-Value	Corrected *p*-Value*	Controls *n* = 38	*p*-Value	Corrected *p*-Value *
Anti-skin antibodies, U/mL								
DSG1	3.83 (0.18–38.83)	2.67 (0.18–20.2)	5.62 (1.25–38.83)	0.022	0.066	2.12 (0–15.09)	0.331	0.382
DSG3	3.72 (0–40.4)	2.8 (0–10.02)	6.14 (0.94–40.4)	<0.001	<0.001	1.58 (0.08–8.38)	0.101	0.152
BP180	7.1 (0.5–118.8)	5.7 (0.5–83.3)	14.2 (2.2–118.8)	0.005	0.019	1.82 (0–34.41)	0.015	0.038
BP230	4.75 (0.5–66)	3.7 (0.5–15.6)	12.7 (1.97–66)	0.003	0.015	1.68 (0.22–26.74)	0.02	0.043
COL7	1.92 (0–41.05)	1.08 (0–7.58)	4.96 (0.3–41.05)	<0.001	<0.001	0.26 (0–3.99)	<0.001	<0.001
Cytokines, pg/mL								
IL-1β	16.62 (1.16–192.67)	8.03 (1.16–144.31)	37.74 (2.23–192.67)	0.101	0.216	2.75 (0.88–189.66)	0.009	0.027
IL-2	111.23 (18.76–3992.66)	105.15 (18.76–3992.66)	120.7 (34.86–3475.02)	0.548	0.685	31.61 (8.01–2400)	0.002	0.008
IL-4	0.96 (0.79–4.5)	0.96 (0.79–4.5)	0.96 (0.79–3.26)	0.39	0.65	0.94 (0.6–2.07)	0.153	0.202
IL-6	33.44 (5.32–1000)	23.77 (5.32–1000)	40.37 (9.28–858.09)	0.086	0.215	8.54 (4.46–1000)	0.002	0.008
IL-8	45.09 (13.14–449)	44 (13.14–449)	49.7 (21.97–124.24)	0.617	0.712	27.96 (12.96–939.06)	0.471	0.505
IL-10	32.98 (8–250)	42.59 (8–250)	30.95 (12.96–187.6)	0.969	0.969	21.12 (5.86–250)	0.034	0.06
IL-12	1.52 (1.14–9.21)	1.44 (1.14–9.21)	1.65 (1.22–3.8)	0.493	0.672	1.36 (1–7.29)	0.162	0.202
TNF-α	175.12 (137.66–4800)	165.29 (137.66–1364.65)	176.27 (148.9–4800)	0.363	0.65	176.1 (128.17–1351.09)	0.648	0.648
TNF-β	1737.07 (1101.8–36,000)	1753.29 (1101.8–36,000)	1719.14 (1149.51–36,000)	0.75	0.804	1267.11 (802.84–3764.79)	<0.001	<0.001
IFN-γ	330.16 (79.18–5000)	279.12 (79.18–5000)	360.25 (110.6–4607.18)	0.468	0.672	176.34 (73.56–4910)	0.036	0.06

BP180: antibodies against bullous pemphigoid 180; BP230: antibodies against bullous pemphigoid 230; COL7: type VII collagen; DEB: dystrophic epidermolysis bullosa; DSG1: antibodies against desmoglein 1; DSG3: antibodies against desmoglein 3; EB: inherited epidermolysis bullosa; IFN: interferon; IL: interleukin; RDEB: recessive dystrophic inherited epidermolysis bullosa; TNF: tumor necrosis factor. Median values (range in parentheses). * *p*-Values were adjusted for multiple testing using the false discovery rate method (with the Benjamini-Hochberg procedure). Controls were compared with other EB types.

**Table 3:** Comparison between generalized and localized inherited epidermolysis bullosa types and healthy controls.

Parameter	Total EB Cases *n* = 42	Localized EB *n* = 18	Generalized EB *n* = 24	*p*-Value	Corrected *p*-Value*	Controls *n* = 38	*p*-Value	Corrected *p*-Value *
Anti-skin antibodies, U/mL								
DSG1	3.83 (0.18–38.83)	2.33 (0.18–20.2)	5.54 (1.1–38.83)	0.016	0.048	2.12 (0–15.09)	0.629	0.629
DSG3	3.72 (0–40.4)	2.56 (0–35.13)	5.19 (0.94–40.4)	0.007	0.026	1.58 (0.08–8.38)	0.266	0.348
BP180	7.1 (0.5–118.8)	3.65 (0.–26.3)	13.37 (2.4–118.8)	0.001	0.005	1.82 (0–34.41)	0.073	0.137
BP230	4.75 (0.5–66)	3 (0.5–15.6)	8.1 (1.9–66)	<0.001	<0.001	1.68 (0.22–26.74)	0.122	0.183
COL VII	1.92 (0–41.05)	0.96 (0–8.2)	4.92 (0.51–41.05)	<0.001	<0.001	0.26 (0–3.99)	0.01	0.036
Cytokines, pg/mL								
IL-1β	16.62 (1.16–192.67)	10.49 (1.16–180.69)	21.07 (1.43–192.67)	0.332	0.553	2.75 (0.88–189.66)	0.019	0.048
IL-2	111.23 (18.76–3992.66)	87.12 (18.76–3992.66)	116.53 (24.76–3475.02)	0.792	0.927	31.61 (8.01–2400)	0.004	0.03
IL-4	0.96 (0.79–4.5)	0.99 (0.79–4.5)	0.96 (0.79–3.26)	0.729	0.927	0.94 (0.6–2.07)	0.278	0.348
IL-6	33.44 (5.32–1000)	14.98 (5.32–1000)	39.44 (9.73–858.09)	0.091	0.227	8.54 (4.46–1000)	0.012	0.036
IL-8	45.09 (13.14–449)	42.9 (13.14–449)	51.44 (13.61–124.24)	0.307	0.553	27.96 (12.96–939.06)	0.426	0.467
IL-10	32.98 (8–250)	66.29 (8–250)	32.17 (12.96–187.6)	0.865	0.927	21.12 (5.86–250)	0.102	0.17
IL-12	1.52 (1.14–9.21)	1.73 (1.14–9.21)	1.44 (1.16–7.14)	0.165	0.354	1.36 (1–7.29)	0.026	0.056
TNF-α	175.12 (137.66–4800)	167.75 (145.89–1364.65)	184.22 (137.66–4800)	0.816	0.927	176.1 (128.17–1351.09)	0.436	0.467
TNF-β	1737.07 (1101.8–36,000)	1719.99 (1101.8–36,000)	1753.29 (1149.51–36,000)	1	1	1267.11 (802.84–3764.79)	<0.001	<0.001
IFN-γ	330.16 (79.18–5000)	357.05 (79.18–5000)	330.16 (110.6–4607.18)	0.85	0.927	176.34 (73.56–4910)	0.011	0.036

BP180: antibodies against bullous pemphigoid 180; BP230: antibodies against bullous pemphigoid 230; COL7: type VII collagen; DSG1: antibodies against desmoglein 1; DSG3: antibodies against desmoglein 3; EB: inherited epidermolysis bullosa; IFN: interferon; IL: interleukin; TNF: tumor necrosis factor. Median values (range in parentheses). * *p*-Values were adjusted for multiple testing using the false discovery rate method (with the Benjamini-Hochberg procedure). Controls were compared with localized EB.

**Table 4:** Comparison of the studied variables in inherited epidermolysis bullosa patients according to their BEBS score and controls.

Parameter	Total IEB Cases *n* = 42	EB Patients with Low-Medium BEBS Score *n* = 28	EB Patients with High BEBS Score *n* = 14	*p*-Value	Corrected *p*-Value *	Controls *n* = 38	*p*-Value	Corrected *p*-Value *
Anti-skin antibodies, U/mL								
DSG1	3.83 (0.18–38.83)	2.6 (0.18–20.2)	6.22 (2.18–38.83)	0.002	0.008	2.12 (0–15.09)	0.291	0.312
DSG3	3.72 (0–40.4)	2.84 (0–35.13)	5.45 (0.94–40.4)	0.004	0.012	1.58 (0.08–8.38)	0.02	0.033
BP180	7.1 (0.5–118.8)	5.9 (0.5–26.3)	23.3 (3.3–118.8)	0.001	0.005	1.82 (0–34.41)	0.007	0.018
BP230	4.75 (0.5–66)	3.65 (0.5–15.6)	13.7 (1.97–66)	<0.001	<0.001	1.68 (0.22–26.74)	0.012	0.022
COL VII	1.92 (0–41.05)	1.08 (0–8.2)	7.39 (0.72–41.05)	<0.001	<0.001	0.26 (0–3.99)	<0.001	<0.001
Cytokines, pg/mL								
IL-1β	16.62 (1.16–192.67)	7.71 (1.16–180.69)	46.78 (3.78–192.67)	0.038	0.081	2.75 (0.88–189.66)	0.005	0.015
IL-2	111.23 (18.76–3992.66)	96.42 (18.76–3992.66)	273.24 (34.86–3475.02)	0.238	0.397	31.61 (8.01–2400)	0.001	0.004
IL-4	0.96 (0.6–4.5)	0.96 (0.6–4.5)	0.93 (0.79–3.26)	0.727	0.875	0.94 (0.6–2.07)	0.231	0.289
IL-6	33.44 (5.32–1000)	16.99 (5.32–1000)	50.77 (17.23–858.09)	0.009	0.022	8.54 (4.46–1000)	0.001	0.004
IL-8	44.3 (4.4–449)	47.4 (4.4–449)	42.18 (21.97–124.24)	0.927	0.927	27.96 (12.96–939.06)	0.282	0.312
IL-10	32.98 (8–250)	44.75 (8–250)	30.95 (17–187.6)	0.817	0.875	21.12 (5.86–250)	0.028	0.038
IL-12	1.52 (1.14–9.21)	1.56 (1.14–9.21)	1.4 (1.22–2.34)	0.157	0.294	1.36 (1–7.29)	0.026	0.038
TNF-α	175.12 (137.66–4800)	167.75 (137.66–1364.65)	198.13 (142.5–4800)	0.497	0.683	176.1 (128.17–1351.09)	0.657	0.657
TNF-β	1737.07 (1101.8–36,000)	1737.07 (1101.8–36,000)	1717.87 (1149.51–36,000)	0.787	0.875	1267.11 (802.84–3764.79)	<0.001	<0.001
IFN-γ	330.16 (79.18–5000)	303.38 (79.18–5000)	387.95 (110.6–4607.18)	0.501	0.683	176.34 (73.56–4910)	0.01	0.021

BEBS: Birmingham Epidermolysis Bullosa Severity score; BP180: antibodies against bullous pemphigoid 180; BP230: antibodies against bullous pemphigoid 230; COL7: type VII collagen; DSG1: antibodies against desmoglein 1; DSG3: antibodies against desmoglein 3; EB: inherited epidermolysis bullosa; IFN: interferon; IL: interleukin; TNF: tumor necrosis factor. Median values (range in parenthesis). * *p*-Values were adjusted for multiple testing using the false discovery rate method (with the Benjamini-Hochberg procedure). Controls were compared with EB patients with a low-to-medium BEBS score.

**Table 5:** Correlation between anti-skin antibodies, serum cytokine levels, and the Birmingham Epidermolysis Bullosa Severity (BEBS) score in inherited epidermolysis bullosa patients.

Parameter	Spearman *p*	*p*-Value	Corrected *p*-Value *
Anti-skin antibodies, U/mL			
DSG1	0.32	0.039	0.084
DSG3	0.331	0.032	0.08
BP180	0.449	0.003	0.015
BP230	0.507	0.001	0.008
COL VII	0.588	<0.001	<0.001
Cytokines, pg/mL			
IL-1β	0.208	0.187	0.351
IL-2	0.064	0.688	0.794
IL-4	−0.156	0.331	0.485
IL-6	0.401	0.008	0.03
IL-8	−0.127	0.43	0.636
IL-10	0.135	0.402	0.548
IL-12	−0.378	0.018	0.054
TNF-α	0.145	0.359	0.538
TNF-β	−0.015	0.926	0.926
IFN-γ	−0.026	0.868	0.926

BP180: antibodies against bullous pemphigoid 180; BP230: antibodies against bullous pemphigoid 230; COL7: type VII collagen; DSG1: antibodies against desmoglein 1; DSG3: antibodies against desmoglein 3; EB: inherited epidermolysis bullosa; IFN: interferon; IL: interleukin; TNF: tumor necrosis factor. Median values (range in parentheses). * *p*-Values were adjusted for multiple testing using the false discovery rate method (with the Benjamini-Hochberg procedure).

**Table 6:** Correlation between interleukin (IL)-6 serum level and anti-skin antibodies in inherited epidermolysis bullosa patients.

Parameter	Spearman *p*	*p*-Value	Corrected *p*-Value *
Anti-skin antibodies, U/mL			
DSG1	0.402	0.008	0.015
DSG3	0.349	0.023	0.023
BP180	0.355	0.021	0.023
BP230	0.397	0.009	0.015
COL VII	0.508	0.001	0.005

BP180: antibodies against bullous pemphigoid 180; BP230: antibodies against bullous pemphigoid 230; COL7: type VII collagen; DSG1: antibodies against desmoglein 1; DSG3: antibodies against desmoglein 3. * *p*-Values were adjusted for multiple testing using the false discovery rate method (with the Benjamini-Hochberg procedure).
